# Effects of cereal fiber on leptin resistance and sensitivity in C57BL/6J mice fed a high-fat/cholesterol diet

**DOI:** 10.3402/fnr.v60.31690

**Published:** 2016-08-16

**Authors:** Ru Zhang, Jun Jiao, Wei Zhang, Zheng Zhang, Weiguo Zhang, Li-Qiang Qin, Shu-Fen Han

**Affiliations:** 1 Jiangsu Key Laboratory of Preventive and Translational Medicine for Geriatric Disease, Department of Nutrition and Food Hygiene, School of Public Health, Soochow University, Suzhou, China; 2 DSM Nutritional Products Human Nutrition and Health, Beijing, China

**Keywords:** cereal fiber, leptin resistance, leptin signaling, high-fat/cholesterol diet

## Abstract

**Background:**

Cereal fiber is reported to be associated with obesity and metabolic diseases. However, whether cereal fiber improves leptin resistance and sensitivity remains unclear.

**Design:**

For 24 weeks, 48 male C57BL/6J mice were randomly given a normal chow diet (Chow), high-fat/cholesterol diet (HFD), HFD with 0.8% oat fiber (H-oat) or HFD with 0.8% wheat bran fiber (H-wheat). At the end of feeding period, both the serum insulin and leptin levels were determined by ELISA kits. Western blotting was used to assess the protein expressions of the leptin receptor (LepR) and the leptin-signaling pathway in the adipose tissues.

**Results:**

Our results suggested that mice fed oat or wheat bran fiber exhibited lower body weight, serum lipids, as well as insulin and leptin levels. The two cereal fibers potently increased the protein expressions of LepR in the adipose tissue. In addition, protein expressions of Janus kinase 2 (JAK2) and transcription 3 (STAT3) (induced by LepR), which enhances leptin signaling, were significantly higher and the expression of cytokine signaling-3 (SOCS3), which inhibits leptin signaling, was significantly lower in the two cereal fiber groups than in the HFD group.

**Conclusion:**

Taken together, our findings suggest that cereal fiber can improve leptin resistance and sensitivity by the JAK2/STAT3 pathway in C57BL/6J mice fed a HFD; furthermore, oat fiber is more effective in the improvement of leptin sensitivity than wheat bran fiber, in this murine model.

Leptin, a peptide hormone mainly secreted by adipocytes, plays a vital role in body weight regulation by suppressing food intake and increasing energy expenditure (1). The effect of leptin on food intake is mediated in part via leptin receptors (LepRs) presented in the hypothalamus. Peripherally applied leptin in rodents induces a central neuronal signaling pathway that involves the activation of a signal transducer and activator of transcription 3 (STAT3) (2). The requirement of this pathway to prevent severe hyperphagia and obesity was recently demonstrated in mice specifically lacking the STAT3-binding site of the LepR (3). After binding to the long LepR, STAT3 becomes phosphorylated by Janus kinase 2 (JAK2) and acts in the nucleus to regulate transcription (4). On the other hand, signaling molecules, such as cytokine signaling-3 (SOCS3), mitigate leptin actions via tyrosine 1,138 of LepR in hypothalamic neurons (5). Thus, a negative feedback mechanism exists in leptin-induced STAT3 signaling, through the induction of SOCS3 (6). Exogenous leptin administration failed as an effective approach to manage obesity, even though therapies that improve leptin sensitivity have become one of the developing alternative approaches to treat obesity and related comorbidities (7, 8).

Cereal fiber has been linked to the prevention of a number of obesity-associated diseases and disorders by decreasing appetite and weight gain (9, 10). Recently, several studies have focused on the metabolic benefits of cereal fiber supplementation, such as body weight management and the improvement of insulin resistance (11, 12). Animal and population studies have demonstrated that fiber can reduce plasma leptin and fiber intake and is inversely associated with plasma leptin concentrations (13, 14). However, the effects of cereal fiber supplementation on leptin resistance and leptin sensitivity remain unclear. Therefore, the aim of the present study was to explore the effects of cereal fiber, including those of oat and wheat bran, on leptin sensitivity by the mechanism of the JAK2/STAT3 signaling pathway in the adipose tissue of mice.

## Materials and methods

### The treatment of the animals and experiment design

A total of 48 7-week-old male C57BL/6J mice were obtained from SLAC Laboratory Animal Company and housed in an air-conditioned environment (22±2°C) with 60% humidity and a 12-h light–dark cycle. After 14 days of acclimatization, animals were randomly allocated to the following four dietary groups (12 mice in each group): the chow diet (Chow) group, the high-fat/cholesterol diet (HFD) group, the HFD plus 0.8% oat fiber (H-oat) group, and the HFD plus 0.8% wheat bran fiber (H-wheat) group. Chow (3.90 kcal/g) was purchased from Research diets Inc., which contained 11.5% fat, 67.7% carbohydrates, and 20.8% protein. HFD (4.77 kcal/g) was also obtained from Research diets Inc., which contained 46% fat, 34.4% carbohydrates, and 19.6% protein. Oat fiber (OatWell^®^22) was granted from DSM Nutritional Products Ltd. Wheat bran fiber was obtained from Shanxi Aote Food Science and Technology Company. Dietary fiber was directly mixed with HFD according the above recipe. The animals were allowed access to food and water during the whole experiment *ad lib*. The experiment lasted for 24 weeks. All of the animal studies were treated in accordance with the Guidelines in the Care and Use of Animals and with the approval of the Soochow University Animal Welfare Committee. All possible efforts were made to minimize the suffering and the number of animals used in the present study.

### Body weight, serum lipids, and insulin and leptin levels

During the whole experiment, body weights and food intake were recorded weekly. Energy intake was calculated according to food intake and calories content. Blood samples were taken from the retrobulbar vein of fasted mice (12–14 h) at the start, 8th, 16th and 24th week of the diet study. Serum was separated by centrifugation, and stored at −80°C freezer until assayed. After the mice were sacrificed, epididymal adipose tissues were quickly removed and stored at −80°C freezer for further analysis. Serum total cholesterol and triglycerides were determined by enzymatic methods using commercial kits (Applygen Technologies Inc., Beijing, China). Serum insulin and leptin levels were measured by commercial ELISA kits, according to strict specifications (Mercodia, Sweden and Merck Millipore Bioscience, CA, USA). The appropriate detection range for insulin and leptin assay was, respectively, 0.2 µg/L to 6.5 µg/L, 0.23 ng/mL to 30 ng/mL, and the lowest detection level was, respectively, 0.2 µg/L and 0.05 ng/mL.

### Western blot analysis in the adipose tissues

Epididymal adipose tissue samples were homogenized in lysis buffer (Beyotime Institute of Biotechnology, Nantong, China) and centrifuged at 13,000 g for 15 min. After collecting the supernatants, the protein concentration was determined, according to the BCA protein assay (Beyotime Institute of Biotechnology, Nantong, China). Equal amounts of protein (50–100 µg) were loaded into 12% sodium dodecyl sulfate-polyacrylamide gel and then transferred to a polyvinylidene difluoride membrane by electrophoretic transfer. Blots were incubated overnight at 4°C with the following primary antibodies: leptin (1:2,000, Abcam), LepR (1:2,000, ImmunoWay), JAK2 (1:1,000, Cell Signaling Technology), STAT3 (1:1,000, Cell Signaling Technology), and SOCS3 (1:1,000, Cell Signaling Technology). Subsequently, blots were washed three times and antigen-antibody complexes were incubated for 1 h at room temperature with Peroxidase AffiniPure goat anti-mouse or anti-rabbit IgG antibody (1:2,000, Jackson ImmunoResarch Laboratories). Antibody reactivity was detected by chemiluminescene ECL Detection Systems (EMD Millipore). To confirm the reproducibility of results, each blot was performed at least three times. The intensity of the bands was normalized, using each corresponding β-actin density as an internal control.

### Statistical analysis

All statistical parameters were calculated using an SPSS version 17.0 statistical analysis package (SPSS Inc., Chicago, IL, USA) and expressed as a mean and standard deviation (SD). One-way ANOVA was used to analyze differences among groups, followed by the LSD post hoc test. A *p<*0.05 was considered statistically significant.

## Results

**Fig. 1:**
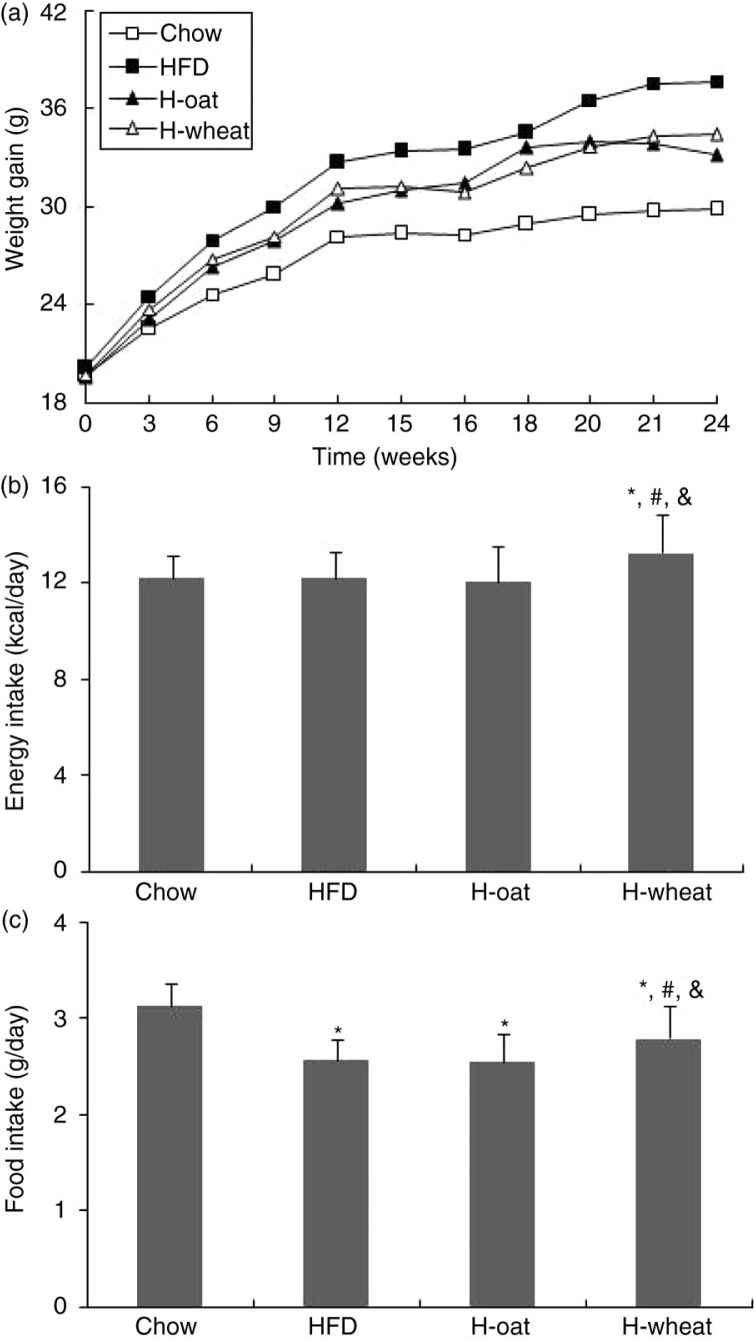
Changes in body weights (a), the food intake (b), and the energy intake (c) of mice in the four diet groups during the whole experiment. Data are means for 12 mice with SD represented by vertical bars. The mean value was significantly different from that of the chow group at *p<0.05, from that of the HFD group at ^#^*p*<0.05, and from that of the H-oat group at ^&^*p*<0.05. HFD, high-fat diet; H-oat, high-fat diet supplemented with 0.8% oat fiber; H-wheat, high-fat diet supplemented with 0.8% wheat bran fiber.

After 24 weeks’ feeding, a trend of increasing body weights was observed in the four different diet groups. At the end of the 24 weeks’ feeding experiment, body weights did not differ between the two groups of H-oat and H-wheat, but were much lower in the HFD group (*p*<0.01) (Fig. 1a). Interestingly, the H-wheat group tended to have higher average food intake and energy intake compared with the two HFD and H-oat groups during the whole experiment (all *p*<0.05) (Fig. 1b and c).

**Fig. 2:**
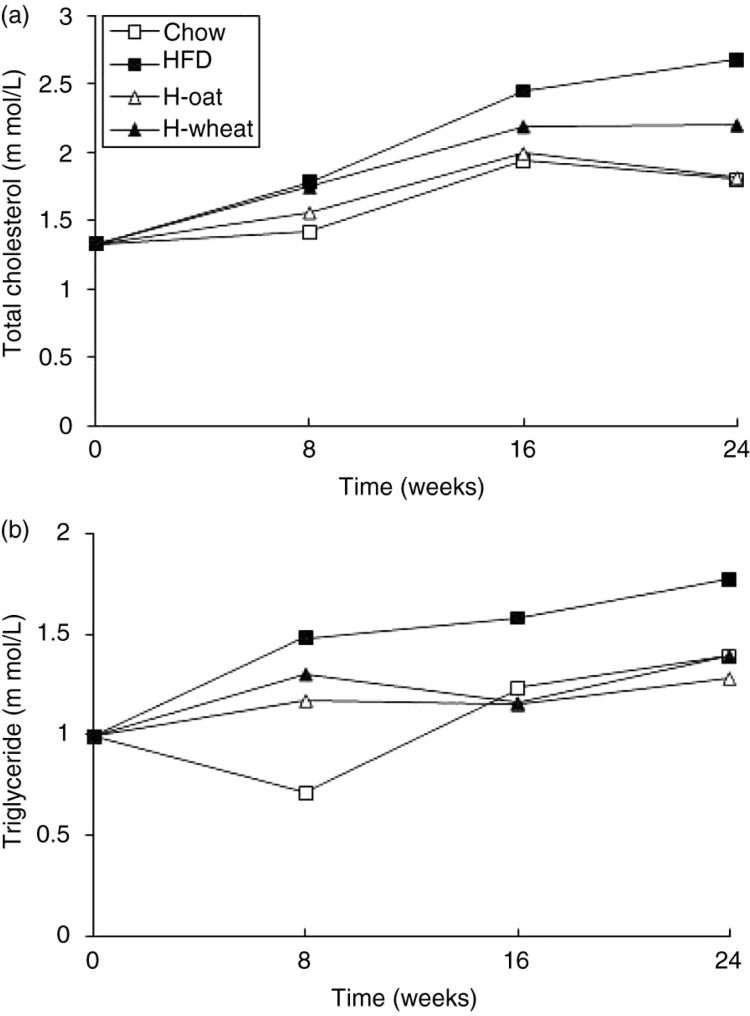
Changes in the total cholesterol (a) and triglycerides (b) of mice in the four diet groups during the whole experiment. Data are the means for 10~12 mice with SD represented by vertical bars. HFD, high-fat diet; H-oat, high-fat diet supplemented with 0.8% oat fiber; H-wheat, high-fat diet supplemented with 0.8% wheat bran fiber.

**Fig. 3:**
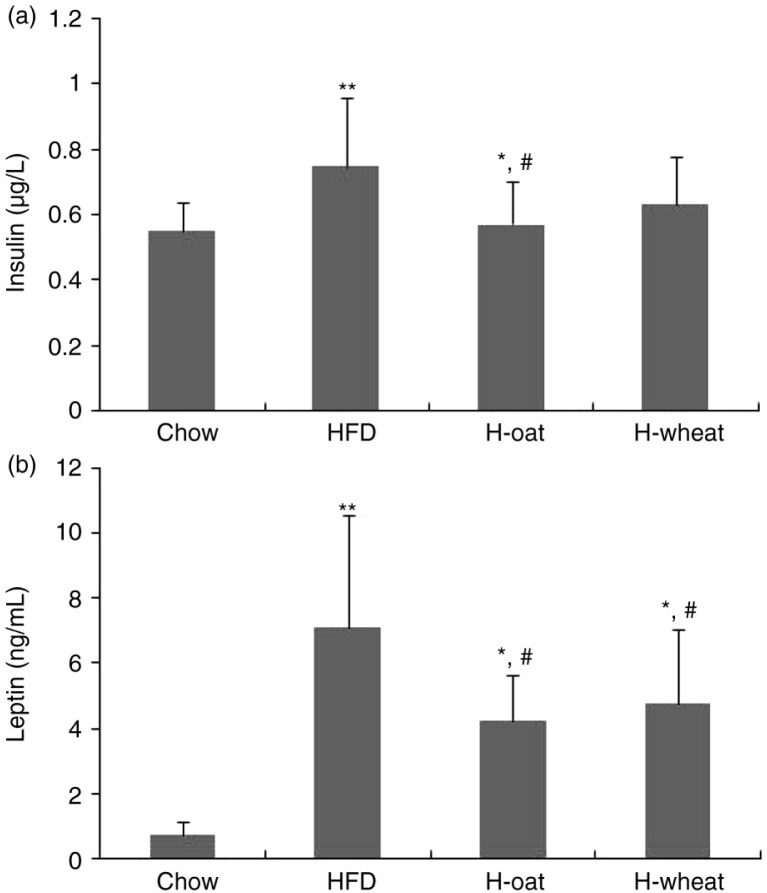
Changes in the serum leptin (a) and insulin (b) of mice in the four diet groups during the whole experiment. Data are means for 12 mice with SD represented by vertical bars. The mean value was significantly different from that of the Chow group at **p<0.01, and from that of the HFD group at ^#^ *p*<0.05. HFD, high-fat diet; H-oat, high-fat diet supplemented with 0.8% oat fiber; H-wheat, high-fat diet supplemented with 0.8% wheat bran fiber.

Ten animals from 48 mice were randomly selected to measure serum total cholesterol and triglycerides as a baseline. After 24 weeks’ feeding, compared with the HFD group, H-oat and H-wheat decreased the serum total cholesterol and triglycerides concentration (all *p*<0.05) (Fig. 2a and b). As shown in Fig. 3, serum insulin and leptin levels were also measured. Compared with the Chow-fed mice, the HFD-fed mice exhibited a significant increase in serum insulin and leptin levels. Compared with the HFD group, the serum insulin level was significantly lower in the H-oat group, and serum leptin levels were significantly lower in the two fiber groups of H-oat and H-wheat (all *p<*0.05). No significant difference was observed in the serum insulin of the HFD and H-wheat groups (*p*>0.05). Compared with the H-wheat group, concentrations of serum insulin or leptin showed a decreasing trend in the H-oat group, but there were no significant differences in the two groups (all *p>*0.05).

**Fig. 4:**
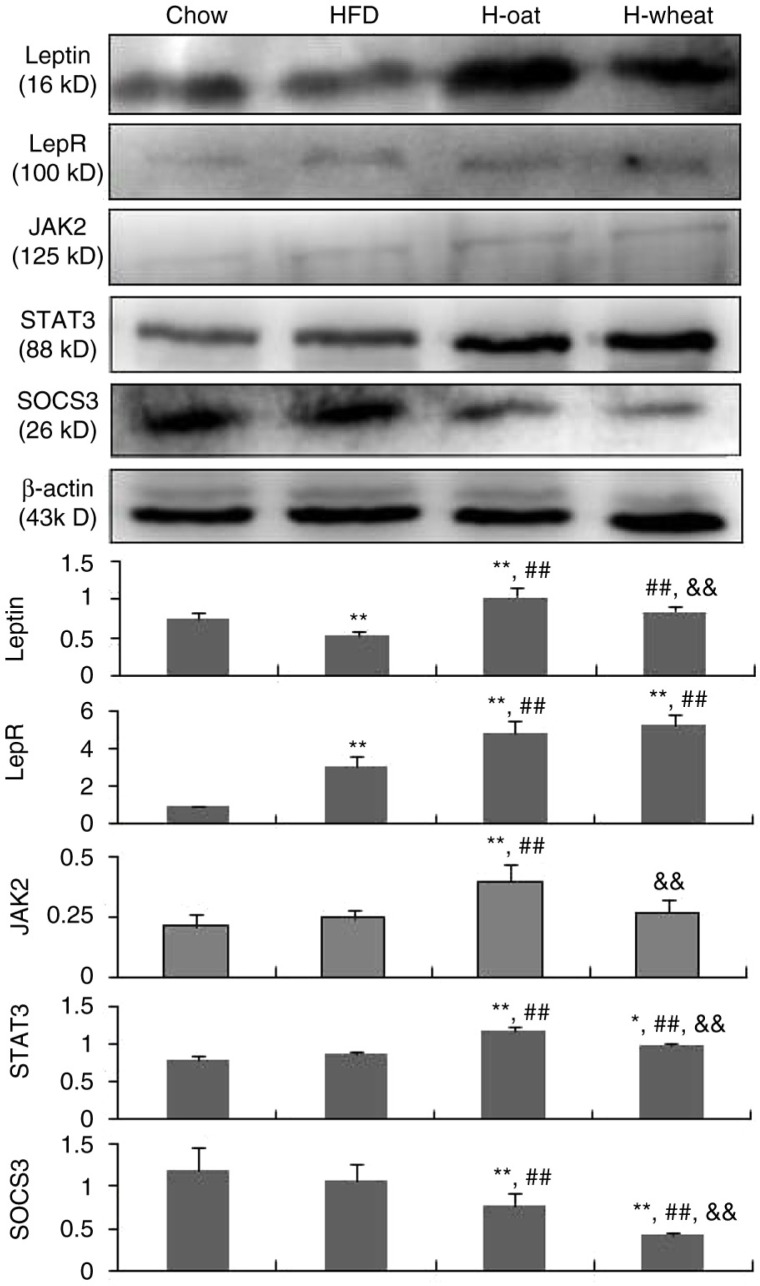
The relative protein expression of leptin, LepR, JAK2, STAT3, and SOCS3 in the four diet groups. The intensity of the bands was quantified by densitometric analysis and normalized with corresponding β-actin. Data are means for six mice with SD represented by vertical bars. The mean value was significantly different from that of the Chow group at *p<0.05 or **p<0.01, from that of the HFD group at ^##^ *p*<0.01, and from that of the H-oat group at ^&&^ *p*<0.01. HFD, high-fat diet; H-oat, high-fat diet supplemented with 0.8% oat fiber; H-wheat, high-fat diet supplemented with 0.8% wheat bran fiber. HFD, high-fat diet; H-oat, high-fat diet supplemented with 0.8% oat fiber; H-wheat, high-fat diet supplemented with 0.8% wheat bran fiber; LepR, leptin receptor; JAK2, Janus kinase 2; STAT3, transcription 3; SOCS3, cytokine signaling-3.

Figure 4 shows the effects of oat fiber and wheat bran fiber on the leptin-signaling pathway in adipose tissues. Compared with the HFD group, the protein expression of leptin, LepR, and STAT3 was higher and that of SOCS3 was lower in the two groups of H-oat and H-wheat (all *p*<0.05), and JAK2 was higher in the H-oat group. In addition, there were significant differences in the expression of these proteins, except LepR, in the two groups of H-oat and H-wheat (all *p*<0.05).

## Discussion

A high cereal fiber intake is emphasized in the recommendations of most nutritional and metabolic associations. In the present study, we found that cereal fiber from oat or wheat bran improved leptin resistance and sensitivity by increasing the protein expressions of JAK2 and STAT3 (which sensitizes the leptin signaling), and by decreasing the protein expression of SOCS3 (which blunts the leptin signaling). In addition, the effects of oat fiber on JAK2/STAT3 signaling were more significant than those of wheat bran fiber.

Our present findings showed that long-term HFD resulted in additional body weight gain, and increased serum total cholesterol and triglycerides levels in the C57BL/6J mice, which were similar to the results of previous studies by our laboratory and others (15, 16). These metabolic abnormalities were blunted in the same animals receiving supplementation with oat or wheat bran fiber. Most animal studies also demonstrated the effect of fiber on weight/fat loss and lipid lowering in diet-induced obese mice (12, 15, 16). Interestingly, the averages of both food intake and energy intake in the H-wheat group were much higher than those in the H-oat group, although there was no significant difference in the final body weights between the two groups. These data suggested that wheat bran fiber, rich in insoluble fiber, may have more effect on body weight management than bran fiber, rich in soluble fiber. Isken found that insoluble fiber was more effective than soluble fiber in controlling body weights (15), which is consistent with our current result. Through a dynamic observation, we found a significantly lower effect on serum lipids in the two cereal fiber groups, compared with the effect on the HFD group, after 16 weeks’ feeding. This suggested that a long-time cereal fiber supplementation was positive in its effect on serum lipids, including total cholesterol and triglycerides. This is consistent with the results of animal studies, however, population studies demonstrated that soluble fiber only lowered plasma total cholesterol (17). This decrease may be accounted for a mechanism by which soluble fiber is fermented by intestinal microflora, such that the fermentation could then modify the production of short chain fatty acids, thereby reducing the acetate and increasing the propionate synthesis.

Population studies demonstrated that high fiber intake, particularly soluble fiber, is significantly related to reducing insulin resistance (18). Animal studies demonstrated that soluble fiber decreases the postprandial glucose curve and possibly ameliorates both the resistance and sensitivity to insulin in Zucker Diabetic Fatty rats (11). Our result also showed that a cereal fiber supplement decreased the levels of serum insulin, and oat fiber, rich in soluble fiber, may be better for insulin resistance than wheat bran fiber, rich in insoluble fiber. Serum leptin, which is a cytokine-like hormone predominantly produced in the white adipose tissue and secreted into the systemic circulation, was positively correlated with markers of insulin resistance. Leptin suppresses energy intake and stimulates energy expenditure, leading to a reduction in stored body fat. Some studies indicated that diet-induced obesity may induce leptin resistance, a phenomenon characterized by elevated circulating leptin levels and decreased leptin sensitivity (19). Our results showed that the serum leptin level in the HFD group was the highest, however, after 24-weeks’ feeding with HFD-supplemented cereal fiber, while serum leptin levels were significantly inhibited and leptin resistance and sensitivity improved. Several studies observed the effect of fiber-rich diets on modulating leptin secretion and ameliorating leptin resistance in high-fat-diet-fed mice (12, 16). All in all, these results, based on circulating parameters, suggest that cereal fiber has beneficial metabolic health effects via improving leptin resistance and sensitivity.

Leptin exerts its regulatory effects on energy homeostasis through its receptors (LepR) present in the hypothalamus and corresponding signaling pathways. JAK2/STAT3 signaling is a representative signaling pathway through which leptin regulates food intake and energy homeostasis (3, 20). Our results showed that cereal fiber significantly increased the protein expression of leptin and LepR in the adipose tissues. Besides that, our study also showed that cereal fiber increased the protein expression of JAK2 and STAT3 in the adipose tissues. Leptin can bind to its receptors (LepR) and initiate downstream signaling through the sequential phosphorylation of the tyrosine kinase JAK2 and the transcription factor signal transducer and activators of STAT3. Then the phosphorylation STAT binds DNA and modulates the transcription of genes involved in food intake and energy homeostasis (21). SOCS3 is known to inhibit leptin activities. Thus, a negative feedback mechanism exists in leptin-induced STAT3 signaling, through the induction of SOCS3 (6). A recent research that investigated the role of SOCS3 in the regulation of leptin sensitivity indicated that the inactivation of SOCS3 in LepR-expressing cells protected mice from leptin resistance induced by a high-fat diet (7). In the present study, the protein expression of SOCS3 was dramatically down-regulated by cereal fiber. The results suggested that cereal fiber can improve leptin resistance and sensitivity through the JAK2/STAT3 pathway, which may be vital for the maintenance of energy homeostasis through the fine-tuning of a modulatory loop in the molecular network of leptin action. As far as we know, our study is one of the first to explore the effect of cereal fiber on the JAK2/STAT3 pathway. Considering the differences among cereal fiber types on JAK2/STAT3 pathway, we found that oat fiber was more effective than wheat bran fiber. The difference may result from the differences of molecular structure of cereal fiber. Cereal fiber can be considered a ‘black box’ since its molecular structure can vary significantly (22). The physiological effects of cereal fiber may be related to its molecular structure. However, this issue remains to be elucidated.

In the present study, we did not specifically investigate whether cereal fiber improved leptin resistance in the central nervous system (CNS) and skeletal muscle. However, most of the potential mechanisms underlying leptin resistance have been explored in the CNS (23, 24), and very little has been published regarding leptin resistance in peripheral tissues. It is still controversial whether leptin resistance resides mainly in the CNS or in peripheral tissues (25). Thus, further studies are needed to elucidate whether cereal fiber also effectively improves leptin resistance and sensitivity in the CNS. In addition, the signaling pathways known to mediate the actions of leptin resistance also include PI3K–Akt–FoxO1 signaling, AMP-activated protein kinase (AMPK) signaling, and mTOR–S6K signaling. The precise molecular mechanisms of cereal fiber on leptin resistance and sensitivity in-depth remain to be elucidated, in order to promote the proof that cereal fiber prevents obesity-associated diseases.

In conclusion, the present study suggests that a high cereal-fiber intake can improve HFD-induced leptin resistance and sensitivity through the leptin signal pathway in the adipose tissue. Specifically, cereal fiber increases leptin signaling by enhancing the expression of JAK2 and STAT3 (induced by LepR), and inhibiting the expression of SOCS3. In addition, oat fiber is more effective in the improvement of leptin resistance and sensitivity than wheat bran fiber.
